# Investigating the Relationships Between COVID-19 Cases, Public Health Interventions, Vaccine Coverage, and Mean Temperature in Ontario and Toronto

**DOI:** 10.3390/diseases13080269

**Published:** 2025-08-19

**Authors:** Melinaz Barati Chermahini, Vernon Hoeppner

**Affiliations:** 1 Public Health Sciences Department, Queen’s University, 62 Fifth Field Company Lane, Kingston, ON K7L 3N6, Canada; 2 College of Medicine, University of Saskatchewan, 107 Wiggins Rd, Saskatoon, SK S7N 5E5M, Canada

**Keywords:** vaccine, epidemiology, infectious diseases, COVID-19

## Abstract

**Background/Objectives:** We aimed to examine the relationship between COVID-19 cases and Public Health Interventions (PHIs), vaccine coverage, and temperature. We compared our findings with those of other studies that used different methodologies, such as mathematical models. **Methods**: We developed monthly PHI scores using the Oxford COVID-19 Government Response Tracker from May 2020 to May 2021. We calculated PHI scores by summing the highest monthly score of each intervention and expressed the PHI score as a percentage of the maximum. We obtained vaccine coverage and temperature data from January 2021 to September 2023. We calculated Spearman’s rank-order correlation coefficients to examine correlations. **Results**: The correlation between cases and PHI was positive (ρ = 0.947, *p* < 0.0001). The correlation between cases and vaccine coverage was approximately zero (ρ = 0.0165, *p* = 0.957) from January 2021 to January 2022 and was negative from February 2022 to September 2023 (ρ= −0.816, *p* < 0.0001). The correlation for cases and temperature was negative from January 2021 to January 2022 (ρ = −0.676, *p* = 0.0112) and was almost zero from February 2022 to September 2023 (ρ = −0.162, *p* = 0.494). The models showed a negative correlation between PHI and vaccine coverage, and mixed results for temperature. **Conclusions**: There was a positive correlation between cases and PHI. Prior to reaching the vaccine threshold coverage, there was no correlation for vaccination and a negative correlation for temperature. Post-vaccine threshold, there was a negative correlation for vaccination and no correlation for temperature. Correlation results for PHI and temperature differed from those of the mathematical models.

## 1. Introduction

The first case of COVID-19 in Canada was identified on 25 January 2020, in Ontario [1]. Canada’s response to COVID-19 included federal and provincial public health interventions (PHIs), which are defined as non-pharmaceutical and pharmaceutical measures implemented to minimize serious illness and death, while reducing societal disruptions [2]. The federal government was responsible for several PHIs, such as restricting international travel, providing economic support, and vaccine distribution. Provincial governments implemented other PHIs, including case management, the closure of non-essential businesses, and social distancing.

The relationship between COVID-19 cases and PHIs has been explored in previous studies using mathematical models and correlation analysis over wide geographic regions, with time frames of less than 4 months leading up to May 2020 [3,4,5,6]. Notably, these studies reported a negative relationship between cases and PHIs [7,8]. Moreover, the relationship between cases of other respiratory infections, such as Influenza, and PHIs has been explored. Lagacé-Wiens et al. analyzed the impact of PHIs implemented in March of 2020 in response to COVID-19 cases in Canada on Influenza A and B epidemiology [9]. They compared national laboratory surveillance data obtained over 18 weeks (from 29 December 2019 to 9 May 2020) with 9-year historical influenza season control data. Their findings indicate a decrease in Influenza A and B cases during the PHI implementation period.

Vaccines were pharmaceutical measures implemented by the federal government beginning in December 2020 [10]. By September of 2023, 80.5% of the Canadian population had received two doses of the COVID-19 vaccine [11]. Moreover, only a few studies have explored the relationship between vaccination coverage and cases [12,13]. For instance, Wu et al. [14] used mathematical modeling to show that vaccine effectiveness against infections decreased over time. These studies focus on cross-country data with study periods of less than 19 months. It is important to further explore the impact of vaccination in more localized geographic regions over an extended period.

Temperature has been found to significantly influence the development of respiratory infections [15]. It is important to further explore the impact of temperature on respiratory infections. Previous findings show that low temperatures and low radiation/sunlight lead to better survival of coronaviruses in winter [16]. Identifying the seasonal patterns of viral respiratory infections can inform healthcare systems and facilitate timely public health interventions, thereby protecting populations from infections.

Previous studies have investigated the relationship between COVID-19 cases and temperature [17,18,19]. This relationship has been examined over intervals ranging from 5 weeks to 5 months, utilizing data at country, province, and city levels. Various statistical methodologies, including correlation analysis and mathematical models, have been employed. However, the findings regarding the direction of this relationship are conflicting, as some studies show a negative association [19], whereas others indicate a positive association [18,20].

This study aimed to use correlation analysis to investigate the relationships between reported COVID-19 cases, PHIs, vaccine coverage, and temperature. The relationship between cases and PHIs was explored from May 2020 to May 2021. The relationship between cases and vaccine coverage in Ontario and Toronto temperature was analyzed for the period from January 2021 to September 2023.

## 2. Materials and Methods

### 2.1. Provincial and City Case Data

We gathered data on provincial case numbers for Ontario from open-access datasets available through the Government of Canada’s COVID-19 epidemiology update tool (https://www.canada.ca/en/public-health/services/diseases/2019-novel-coronavirus-infection.html, downloaded 13 April 2024). We used Ontario case data for the PHI and vaccine correlation analysis. We gathered casa data for Toronto from Public Health Ontario’s Respiratory Virus Tool https://www.publichealthontario.ca/en/Data-and-Analysis/Infectious-Disease/Respiratory-Virus-Tool (accessed on 18 September 2023). This open-access dataset includes daily new case counts for each public health unit in Ontario. Moreover, we calculated monthly new case counts for Toronto by summing the weekly new cases reported for the Toronto public health unit each month. We used Toronto case data for temperature correlation.

COVID-19 case data in this study represent individuals who were tested and/or reported to Public Health Units (PHUs) and subsequently recorded in Ontario’s provincial reporting system. Only cases meeting the confirmed case classification as defined by the Ontario Ministry of Health (MOH) were included. This classification includes laboratory-confirmed SARS-CoV-2 infections identified by validated nucleic acid amplification testing (NAAT), point-of-care NAAT, serological evidence of infection, or confirmation through coroner reports where COVID-19 was a cause or contributing factor to death [21].

The datasets used to extract information on cases did not include information on false positives or negatives. Future studies incorporating test sensitivity and specificity could provide valuable insights to better understand case trends.

### 2.2. PHI Data and Score

PHIs are federal and provincial measures implemented to address rising case numbers. Our study focused on non-pharmaceutical and vaccination interventions. We obtained PHI data from the Canadian Institute for Health Information’s (CIHI) Canadian Dataset of COVID-19 Interventions https://www.cihi.ca/en/canadian-covid-19-intervention-timeline (accessed on 18 September 2023). This dataset provided information on various PHIs, including case management, distancing, travel restrictions, and vaccination.

We scored PHIs based on a framework adapted from the Oxford COVID-19 Government Response Tracker [22]. The time scale used for calculating the PHI score was one month. Moreover, we recorded interventions based on an ordinal or binary scale, with the highest value denoting the most stringent level. We calculated PHI scores, expressed as a percentage, by dividing the sum of the highest scores for each intervention by the maximum possible score. Table 1 demonstrates an example of the framework used for scoring the workplace closing intervention implemented from March to September 2020. Data demonstrating the scoring framework and calculated PHI scores is provided in Supplementary Table S1. The maximum score represented a scenario in which each intervention was implemented at its most stringent level. When interventions varied in stringency within a month, we used the highest level for score calculation. If there was no change recorded in the stringency of a PHI compared to the previous month, the score for that intervention remained unchanged.

### 2.3. Vaccine Coverage Data

We gathered vaccine coverage data for Ontario from a publicly accessible dataset available through the Government of Canada’s COVID-19 Vaccination Coverage Monitoring dashboard [11]. This dataset included the reported weekly proportion of fully vaccinated individuals for each Canadian province. Fully vaccinated meant receiving two doses of an authorized vaccine. We used the reported proportion for the last week of each month as the monthly vaccination proportion. We used vaccination data for the timeframe from January 2021 to September 2023.

### 2.4. Temperature Data

We used reported temperature data for Toronto, including monthly mean temperatures [23]. We based the temperature data, measured in degrees Celsius, on readings from the Toronto International Airport weather station, covering the period from January 2021 to September 2023. Cases were obtained from Public Health Toronto (Supplementary Table S2).

### 2.5. Statistical Analysis

Case data from Ontario and Toronto did not fulfill the normality assumption of Pearson’s correlation (Shapiro–Wilk test of normality, W = 0.650, *p* < 0.0001). Thus, we conducted the non-parametric correlation analysis, Spearman’s rank-order correlation, to examine the relationships between new cases, PHIs, vaccine coverage, and temperature. Additionally, to examine the delayed effect of PHIs on case counts, we calculated the correlation between cases and PHIs with a one-month lag in cases. To better illustrate the relationship between vaccine coverage and temperature before and after reaching the vaccine coverage threshold, we conducted correlation analyses over two intervals from January 2021 to January 2022 and from February 2022 to September 2023. We conducted the correlation analysis using SAS OnDemand for Academics (SAS Studio 3.8).

## 3. Results

### 3.1. Correlation Between PHI Scores and New Cases in Ontario

**Figure 1:**
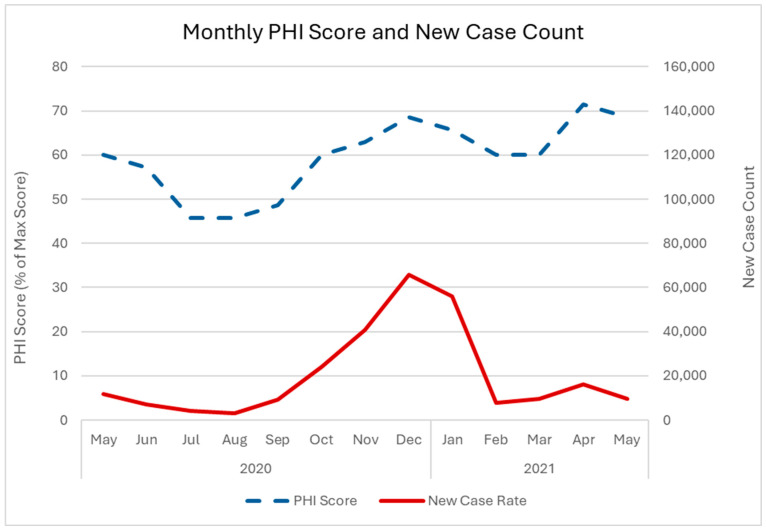
Monthly new COVID-19 case counts and PHI scores as a percentage of the maximum score for Ontario from May 2020 to May 2021. Solid black line = new case counts. Dashed black line = PHI score. We obtained Ontario case data from open access datasets available through the Government of Canada’s COVID-19 epidemiology update tool (https://www.canada.ca/en/public-health/services/diseases/2019-novel-coronavirus-infection.html, downloaded 13 April 2024). The PHI data is from the Canadian Institute for Health Information’s (CIHI) Canadian Dataset of COVID-19 Interventions https://www.cihi.ca/en/canadian-covid-19-intervention-timeline (accessed on 18 September 2023). The PHI scores are calculated based on a framework adapted from the Oxford COVID-19 Government Response Tracker [22].

Figure 1 shows a positive correlation between new cases and PHI score. The first peak in new cases occurred in January 2021, whereas the PHI score peaked in December 2020. The second peak in cases occurred in April 2021, which coincided with the PHI peak. The Spearman’s rank-order correlation was positive and significant (ρ = 0.947, *p* < 0.0001). The correlation analysis with a one-month delay in new cases also showed a positive and significant correlation (ρ = 0.788, *p* = 0.0023).

### 3.2. Correlation Between New Cases and Percent Vaccine Coverage in Ontario

**Figure 2:**
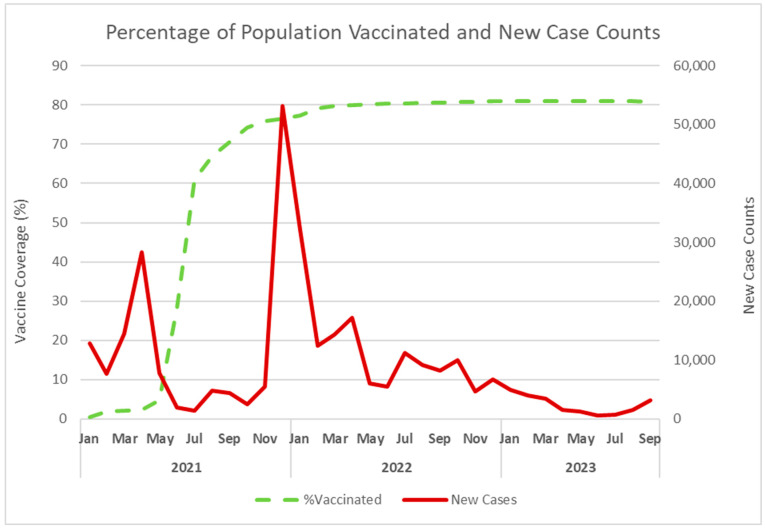
Trend of the monthly percentage of the population vaccinated in Ontario and new case counts in Ontario from January 2021 to September 2023. Solid black line = new case counts. Dashed black line = percentage of population vaccinated. We obtained Ontario case data from open access datasets available through the Government of Canada’s COVID-19 epidemiology update tool (https://www.canada.ca/en/public-health/services/diseases/2019-novel-coronavirus-infection.html, downloaded 13 April 2024). Vaccine coverage data for Ontario is from a publicly accessible dataset, available through the Government of Canada’s COVID-19 Vaccination Coverage Monitoring dashboard. Vaccine proportions for the last week of each month were used as the monthly proportion of the population vaccinated.

The percent of fully vaccinated people increased rapidly from January 2021 to January 2022 (Figure 2). During this period, new case counts decreased during summer, then rose in fall and winter. When vaccine coverage reached 77.3% in January 2022, new cases began decreasing until September 2023.

The correlation between cases and vaccine coverage was approximately zero (ρ = 0.0165, *p* = 0.957) from January 2021 to January 2022, and was negative from February 2022 to September 2023 (ρ = −0.816, *p* < 0.0001).

### 3.3. Correlation Between New Cases and Mean Temperatures in Toronto

**Figure 3:**
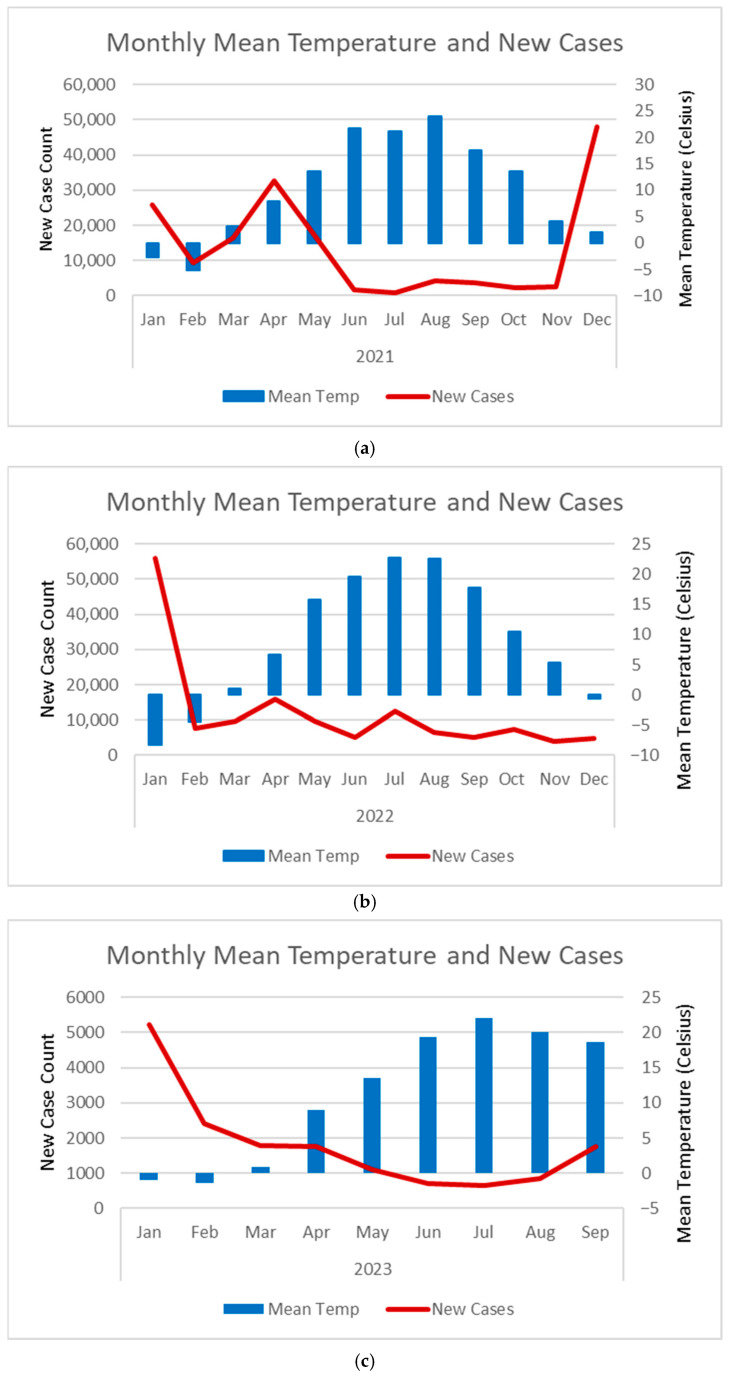
Trend of the monthly mean temperature and new case counts in Toronto for 2021 (**a**), 2022 (**b**), and from January 2023 to September 2023 (**c**). Solid red line = new case counts. Blue bar = mean temperature. The case data for Toronto is from the Public Health Ontario’s Respiratory Virus Tool https://www.publichealthontario.ca/en/Data-and-Analysis/Infectious-Disease/Respiratory-Virus-Tool (accessed on 18 September 2023). We calculated monthly new case counts by summing the weekly new cases reported for Toronto’s public health unit within each month. Temperature data is from Toronto International Airport weather station readings [23].

The correlation coefficient for Toronto’s new cases and the mean temperature from January 2021 to January 2022 was negative and significant (ρ = −0.645, *p* = 0.0112). From February 2022 to September 2023, the correlation approached zero and was not significant (ρ = −0.162, *p* = 0.494). Moreover, Figure 3 shows the trend of monthly mean temperature and case counts for Toronto from January 2021 to September 2023. Case counts were generally lower during the summer months, when temperatures were higher, compared to the winter months, when temperatures fell. This relationship was more evident in 2021 and 2022 (Figure 3a,b).

## 4. Discussion

This study revealed a significant positive correlation between new COVID-19 cases and PHIs. Throughout the study period, we observed that cases and PHIs rose and fell at approximately the same time. Additionally, prior to vaccine coverage reaching the threshold of 77.3%, the relationship between case numbers and vaccine coverage was weak. However, once vaccine coverage exceeded the threshold, a negative and significant correlation was observed between cases and vaccine coverage. Thus, as vaccine coverage increased, cases decreased. Prior to reaching the vaccine coverage threshold, our analysis also found a negative and significant correlation between cases and temperature; therefore, cases were lower during warmer months. After crossing the threshold, the correlation between cases and temperature was almost zero, meaning that the relationship was weak.

The positive correlation between new cases and PHIs contradicts previously published models that show a negative correlation. For instance, Rees et al. [7] investigated the relationship between PHIs and COVID-19 transmission from April 2020 to March 2021 across six Canadian provinces. Similar to our methodology, this study adapted a framework from the University of Oxford OXCGRT Data, https://github.com/OxCGRT/covid-policy-tracker (accessed on 18 September 2023) to calculate the PHI score. They used the effective reproduction number (Rt) as a measure of COVID-19 transmission. Their results showed a negative association between Rt and PHI score. Moreover, Stevens et al. [8] investigated the impact of PHIs on cases and social mobility from April to October 2020. Using regression models, they concluded that mask mandates and higher PHI scores were associated with reduced cases. Cyr et al. [6] investigated the correlation between the days of intervention and the number of reported COVID-19 cases in Canadian provinces, including Ontario, over a three-month period from 25 January to 30 April 2020. Contrary to our study, they did not develop a PHI score and used Pearson’s correlation. Nevertheless, they analyzed similar PHIs and found a positive and significant relationship between cases and PHI duration. Furthermore, Lagacé-Wiens et al. [9] used linear regression analysis to explore the relationship between Canada’s Influenza A and B cases and PHIs during an 18-week intervention period in response to COVID-19. Their findings showed a significant decrease in Influenza A and B cases during this period compared to the control period. We note that different statistical methodologies were employed across the studies referenced above, which may account for discrepancies in findings. Specifically, although our analysis utilizes Spearman’s rank correlation to assess the relationships between cases and PHIs, other studies applied regression analysis and Pearson correlation, which rely on different assumptions about the data. The differences in findings could also be due to differences in the study period and the geographic scope for cases and PHIs.

The positive correlation between reported cases and PHIs suggests that when cases increased, PHIs increased, and when cases decreased, PHIs decreased. This observation does not imply ineffectiveness of PHIs but rather suboptimal timing. The policy implication highlights the importance of establishing a proactive threshold for the implementation of more stringent PHIs at predetermined points when cases begin to rise. Similarly, establishing a proactive threshold for the implementation of less stringent PHIs at a set point for falling cases. Establishing and implementing a proactive threshold for PHIs would not only prevent peaks in cases but also promote a more coordinated response that lessens burden on healthcare services and could minimize societal disruption.

The results show a biphasic correlation between cases and vaccine coverage, namely almost zero correlation until coverage reached 77.3% in January 2022, followed by a negative and significant correlation at higher coverage. Thus, prior to January 2022, the relationship between cases and vaccine coverage was weak and not significant. Once the vaccine coverage threshold of 77.3% was reached, the relationship became significant and as vaccine coverage increased, cases declined. Fetekuni et al. [12] evaluated this association using cross-country data for 87 countries worldwide, including Canada. This study reported a positive Spearman’s rank correlation for Canada, which was not statistically significant. Moreover, Huang et al. [13] used temporal and spatial analysis and concluded that rising vaccination coverage correlated with lower new cases after coverage reached 60%. The global scope and different populations may account for the different vaccination coverage threshold that led to decreasing cases. The policy implications of the results of our study support the approach used by Ontario Public Health, namely, to reach the vaccine coverage threshold as soon as possible.

The results also showed a biphasic correlation between Toronto cases and temperature from May 2020 to September 2023. Initially from May 2020 to January 2022, when vaccine coverage was below the threshold, the correlation was negative and significant, followed by almost zero correlation when vaccine coverage exceeded 77.3%. This suggests that when vaccine coverage is greater than the threshold, vaccines may have a more significant impact on cases compared to temperature.

Previous investigations on the correlation between cases and temperature reported mixed findings. Menebo [20] found a positive but weak correlation between daily cases and the maximum temperature in Oslo, Norway over a 7.6-week interval from 27 February to 2 May 2020. Conversely, Mandal and Panwar [19] found a negative but weak correlation using data from approximately 200 countries over a 3.6-week interval from 25 March to 18 April 2020. Both studies were based on data collected over a short period and did not include variations in temperature, which are observed over an extended study period. A study conducted by To et al. [18] explored the relationship between incident cases and mean temperature from January to May 2020 in Ontario, Alberta, British Columbia, and Quebec. Using multiple regression analysis, they found a positive correlation between cumulative incidence and mean temperature that was not significant. As with our analysis of cases and PHIs, we used the Spearman’s rank correlation coefficient to assess the relationship between cases and temperature. In contrast, some referenced studies (e.g., Menebo and Mandal and Panwar) relied on Pearson’s correlation or regression analyses, which use different measures and rely on different data assumptions. These methodological differences should be considered when interpreting discrepancies between studies. It is also important to note differences in the duration of the study period and larger geographic regions.

### Strengths and Limitations

The strength of our study is centered on methodology, including an extended study period, temperature data over a smaller geographic area, PHI score calculation, and reported cases. Other studies evaluated the relationships using shorter study periods and wider geographic regions. Localized case, PHI, temperature, and vaccine coverage data were used by our study to better account for geographic variation in these variables. Cases were based on provincial and public health unit reports to ensure reliability and accuracy. The one-year study period for investigating the relationship between cases and PHIs included three case peaks, an interval during which vaccine coverage was less than 5% and therefore not affected by vaccine coverage. This extended interval facilitated the observation of the correlation between cases and a broader range of PHIs. The PHI score was calculated as a percentage of the maximum score for each month. This standardized calculation allowed for PHI score comparisons by month. Additionally, our study provided evidence of the relationship between cases and vaccination and temperature over a 32-month period, substantially longer than previous reports. The study period allowed us to examine how the correlations evolved throughout the pandemic. Moreover, the accuracy of a more localized region for temperature, such as the city of Toronto, to determine the correlation between cases and temperature improved the reliability of the results.

Our study also has limitations. The PHI score calculation assigned equal weight to each intervention, not considering potential variation in stringency and the effectiveness of different PHIs. The PHI score was calculated using the same ordinal scale for all interventions, assuming comparable magnitude and the impact of increased stringency. Additionally, public health interventions, such as closures and openings, followed regional implementation. Therefore, during some months, different regions of the province had varying levels of restrictions. The PHI scores calculated for these interventions were based on the highest level of restriction regardless of geographic region. Additional limitations include confounding factors such as compliance, population density, mobility patterns independent of PHIs, age distribution, socioeconomic status, testing rate, and access to testing services. These confounding factors could impact the observed relationship between cases and PHIs, vaccination, and temperature, potentially affecting conclusions about the direction and nature of these relationships. Additionally, our study did not analyze the potential impact of booster vaccination shots and different variants of viruses, such as Omicron, on the relationship between cases and vaccination during the study period. It is essential that future studies collect data on these confounders and account for them in the data analysis.

## 5. Conclusions

This study explored the relationship between cases, PHIs, vaccine coverage, and temperature using provincial public health measures, provincial vaccine coverage, and city temperature from May 2020 to September 2023. The correlation between cases and PHIs was positive and significant. The correlation between cases and vaccine coverage was negative and significant once vaccine coverage exceeded the threshold level of 77.3%. Additionally, the correlation between cases and temperature was negative and significant before vaccine coverage reached 77.3%. Thereafter, the correlation approached zero. These results differ from mathematical models.

## Figures and Tables

**Table 1:** The adapted scoring framework used to obtain the monthly Public Health Intervention (PHI) score for the workplace closing intervention, implemented in Ontario from March to September 2020. PHI data is from the Canadian Institute for Health Information’s (CIHI) Canadian Dataset of COVID-19 Interventions https://www.cihi.ca/en/canadian-covid-19-intervention-timeline (accessed on 18 September 2023). The PHI scores are calculated based on a framework adapted from the Oxford COVID-19 Government Response Tracker [22].

Score	May	June	July	August	September
0 = no measures					
1 = recommend closing or all businesses open with alterations			1	1	1
2 = require closing or working from home for some sectors or categories of workers	2	2			
3 = require closing or working from home for all non-essential workplaces					
Max Score ^a^ (all PHIs Included)	35	35	35	35	35
Actual Score ^b^ (all PHIs Included)	21	20	16	16	17
Actual Score ^c^ (% of MAX)	60.0	57.1	45.7	45.7	48.6

^a^: The max score is the sum of highest possible score for all PHIs in each month. ^b^: The actual score is the sum of the highest score for each PHI implemented in each month. ^c^: The actual score as a percentage of the maximum score was obtained by dividing the actual score by the max score.

## Data Availability

Reported data on COVID-19 cases, PHIs, temperature, and vaccination is publicly available. The sources of these data are outlined in the Section 3 and are cited in the References.

## Supplementary Materials

The following supporting information can be downloaded at: https://www.mdpi.com/article/10.3390/diseases13080269/s1. Table S1: PHI Scoring Framework, Table S2: Temperature Data.

## Abbreviations

The following abbreviations are used in this manuscript:

PHI	Public Health Intervention
