# Supplementary material for: Investigating the Relationships Between COVID-19 Cases, Public Health Interventions, Vaccine Coverage, and Mean Temperature in Ontario and Toronto
Source: Diseases. 2025 Aug 19;13(8):269. doi: 10.3390/diseases13080269 (PMC12385227; doi:10.3390/diseases13080269)
Supplement: Supplementary file 1 [file diseases-13-00269-s001.zip › diseases-3748752 Supplementary Table S2.pdf]

Supplementary Table S2: Monthly Toronto new cases and reported Toronto mean temperatures, measured in degrees Celsius for January 2021 to September 2023. Casa data for Toronto is from Public Health Ontario's Respiratory Virus Tool https://www.publichealthontario.ca/en/Data-and-Analysis/Infectious-Disease/Respiratory-Virus-Tool (accessed on 18 September 2023). We calculated monthly new case counts by summing the weekly new cases reported for Toronto public health unit within each month. Temperature data is from the Toronto International Airport weather station readings [23].

Year	Month	Mean Temp	New Cases
2021	January	-2.7	25801
2021	February	-5.1	9367
2021	March	3.1	16551
2021	April	7.9	32747
2021	May	13.6	17194
2021	June	21.8	1623
2021	July	21.2	807
2021	August	24.0	4227
2021	September	17.6	3584
2021	October	13.5	2392
2021	November	4.2	2569
2021	December	2.1	48036
2022	January	-8.3	55835
2022	February	-4.5	7717
2022	March	1.1	9758
2022	April	6.7	15990
2022	May	15.8	9658
2022	June	19.6	5241
2022	July	22.7	12472
2022	August	22.5	6458
2022	September	17.8	5038
2022	October	10.4	7427
2022	November	5.4	3929
2022	December	-0.6	4964
2023	January	-0.9	5238
2023	February	-1.4	2425
2023	March	0.9	1786
2023	April	9.0	1764
2023	May	13.5	1098
2023	June	19.4	711
2023	July	22.1	646
2023	August	20.1	845
2023	September	18.7	1772
